# The IL-20 Cytokine Family in Rheumatoid Arthritis and Spondyloarthritis

**DOI:** 10.3389/fimmu.2018.02226

**Published:** 2018-09-25

**Authors:** Tue W. Kragstrup, Thomas Andersen, Line D. Heftdal, Malene Hvid, Jens Gerwien, Pallavur Sivakumar, Peter C. Taylor, Ladislav Senolt, Bent Deleuran

**Affiliations:** ^1^Department of Biomedicine, Aarhus University, Aarhus, Denmark; ^2^Department of Rheumatology, Aarhus University Hospital, Aarhus, Denmark; ^3^Department of Clinical Medicine, Aarhus University, Aarhus, Denmark; ^4^Eli Lilly, Herlev, Denmark; ^5^Immuno Oncology Translational Development, Celgene Corportation, Seattle, WA, United States; ^6^Nuffield Department of Orthopedics, Rheumatology and Musculoskeletal Sciences, University of Oxford, Oxford, United Kingdom; ^7^Institute of Rheumatology and Department of Rheumatology, First Faculty of Medicine, Charles University, Prague, Czechia

**Keywords:** cytokine, rheumatoid arthritis, spondyloarthritis, interleukin, IL-10 family, fibroblast, osteoclast, autoantibody

## Abstract

This review describes the IL-20 family of cytokines in rheumatoid arthritis (RA) and spondyloartrhitits (SpA) including psoriatic arthritis. The IL-20 receptor (R) cytokines IL-19, IL-20, and IL-24 are produced in both the peripheral blood and the synovial joint and are induced by Toll-like receptor ligands and autoantibody-associated immune complexes in monocytes. IL-19 seems to have anti-inflammatory functions in arthritis. In contrast, IL-20 and IL-24 increase the production of proinflammatory molecules such as monocyte chemoattractant protein 1 and are associated with bone degradation and radiographic progression. IL-22 is also associated with progression of bone erosions. This suggests that the IL-22RA1 subunit shared by IL-20, IL-22, and IL-24 is important for bone homeostasis. In line with this, the IL-22RA1 has been found on preosteoclasts in early RA. IL-26 is produced in high amounts by myofibroblasts and IL-26 stimulation of monocytes is an important inducer of Th17 cells in RA. This indicates a role for IL-26 as an important factor in the interactions between resident synovial cells and infiltrating leukocytes. Clinical trials that investigate inhibitors of IL-20 (fletikumab) and IL-22 (fezakinumab) in psoriasis and RA have been terminated. Instead, it seems that the strategy for modulating the IL-20 cytokine family should take the overlap in cellular sources and effector mechanisms into account. The redundancy encourages inhibition of more than one cytokine or one of the shared receptors. All IL-20 family members utilize the Janus kinase signaling pathway and are therefore potentially inhibited by drugs targeting these enzymes. Effects and adverse effects in ongoing clinical trials with inhibitors of IL-22 and the IL-22RA1 subunit and recombinant IL-22 fusion proteins will possibly provide important information about the IL-20 subfamily of cytokines in the future.

## Rheumatoid arthritis and spondyloarthritis

### Disease characteristics

Rheumatoid arthritis (RA) and spondyloarthritis (SpA) are both immune-mediated rheumatic diseases characterized by chronic inflammation of the synovial joints. Both inflammatory joint diseases are included in this review as they present distinctive clinical features. RA is characterized by destructive polyarthritis and the involvement of multiple organs (1). SpA covers a group of diseases that affects the joints and entheses including ankylosing spondylitis, psoriatic arthritis, enteropathic arthritis, reactive arthritis, and undifferentiated spondyloarthritis (2). SpA can affect the joints of the axial skeleton and/or the peripheral joints. In both cases, extraarticular involvement is common, e.g., uveitis, inflammatory bowel disease (IBD), psoriasis, or enthesitis. The etiology of both RA and SpA is still largely unknown. In direct comparison, the RA pathogenesis involves more adaptive immune features such as autoreactive B cells and production of the autoantibodies rheumatoid factors (RFs) and anti-cyclic citrullinated peptide (CCP) antibodies, whereas SpA pathogenesis seems to be more driven by lymphocyte subsets producing IL-17A (3). In RA, the ultimate disease manifestation in the joint is bone erosions. These bone erosions are found close to the insertion site of the synovial membrane in connection with the formation of pannus tissue and the presence of osteoclasts (1, 4). In SpA, the typical structural change is new bone formation in the axial skeleton and entheses. However, peripheral joint disease in SpA can be destructive (2, 3, 5). Further, both diseases are associated with the development of osteoporosis because of the inflammatory activation of bone degradation (6).

### Cytokines and chemokines

In the immune system, cytokines are important signaling molecules that coordinate the immune response by mediating the communication between cells through specific receptors. These receptors can be found on cells that traditionally are considered as part of the immune system but also on what historically have been looked upon as non-immune cells such as epithelial cells and fibroblasts. In rheumatic disease, the regulation of cytokines is unbalanced. This involves both insufficient production of inhibitory cytokines and augmented production of proinflammatory cytokines that together contribute to the chronic inflammatory condition. Studying the pathogenesis of the rheumatic diseases has led to the development of biologic disease-modifying antirheumatic drugs (7, 8). TNFα inhibitors were the first validated biological therapy for RA. However, now several other anti-cytokine drugs, lymphocyte-targeting agents and small-molecule inhibitors of signal transduction pathways are available or in clinical trials (9). The purpose of the present review is to describe the role of the IL-20 cytokine family in RA and SpA.

## The IL-20 cytokine family

**Figure 1:**
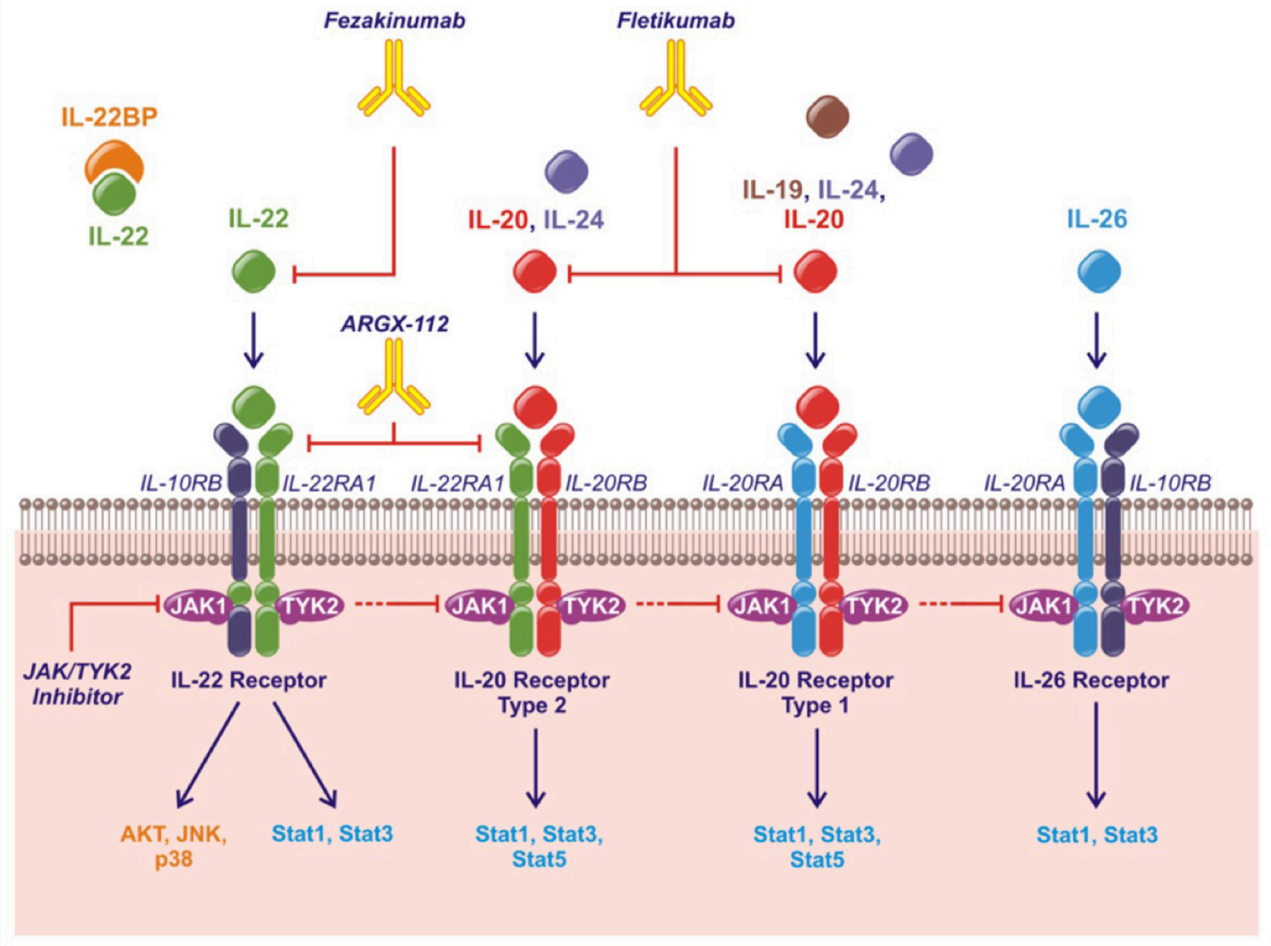
The IL-20 family of cytokines, their shared receptors and intracellular signaling pathways and therapeutic strategies approved or under investigation. Fezakinumab inhibits IL-22. Fletikumab inhibits IL-20. ARGX-112 inhibits the IL-22RA1 subunit. JAK/TYK2 inhibitors will prevent signaling from all the IL-20 family cytokines.

The human IL-20 cytokine family consists of the cytokines IL-19, IL-20, IL-22, IL-24, and IL-26 in the IL-10 superfamily of cytokines (along with IL-10, IL-28, and IL-29) (10, 11). IL-19, IL-20, and IL-24 are also referred to as the IL-20R cytokines based on their shared binding to the receptor complexes containing the IL-20RB. Thus, all three cytokines bind the receptor complex IL-20RB/IL-20RA while only IL-20 and IL-24 also bind the receptor complex IL-20RB/IL-22RA1 (12–14). IL-22 uses the receptor complex IL-10RB/IL-22RA1 and IL-26 signals through IL-10RB/IL-20RA (Figure 1). Murine IL-26 is a pseudogene and the function of mouse IL-24 remains to be elucidated.

The IL-20 cytokine family signal through the Janus kinase (JAK)–signal transducer and activator of transcription (STAT) pathway primarily activating STAT3. Further, IL-22 can activate Akt, extracellular signal-regulated kinases (ERKs), Jun N-terminal kinase (JNK) and p38 mitogen-activated protein kinase (Figure 1) (11).

## IL-19, IL-20, and IL-24

### Expression and regulation

#### Cytokine localization

IL-20 and IL-24 have been identified in mononuclear cells in the synovial membrane of RA patients by immunohistochemistry (15, 16). IL-19 has been found in vimentin+ and CD68+ synovial cells in the hyperplastic lining layers of RA synovial tissues (17). Further, the expression of IL-20 in RA synovial tissue was shown to be particularly associated with macrophages, neutrophil granulocytes and fibroblast-like synovial cells (18). IL-19, IL-20, and IL-24 have all been identified in both synovial fluid and plasma of patients with RA and SpA (15, 16, 18–20).

#### Cellular sources

**Figure 2:**
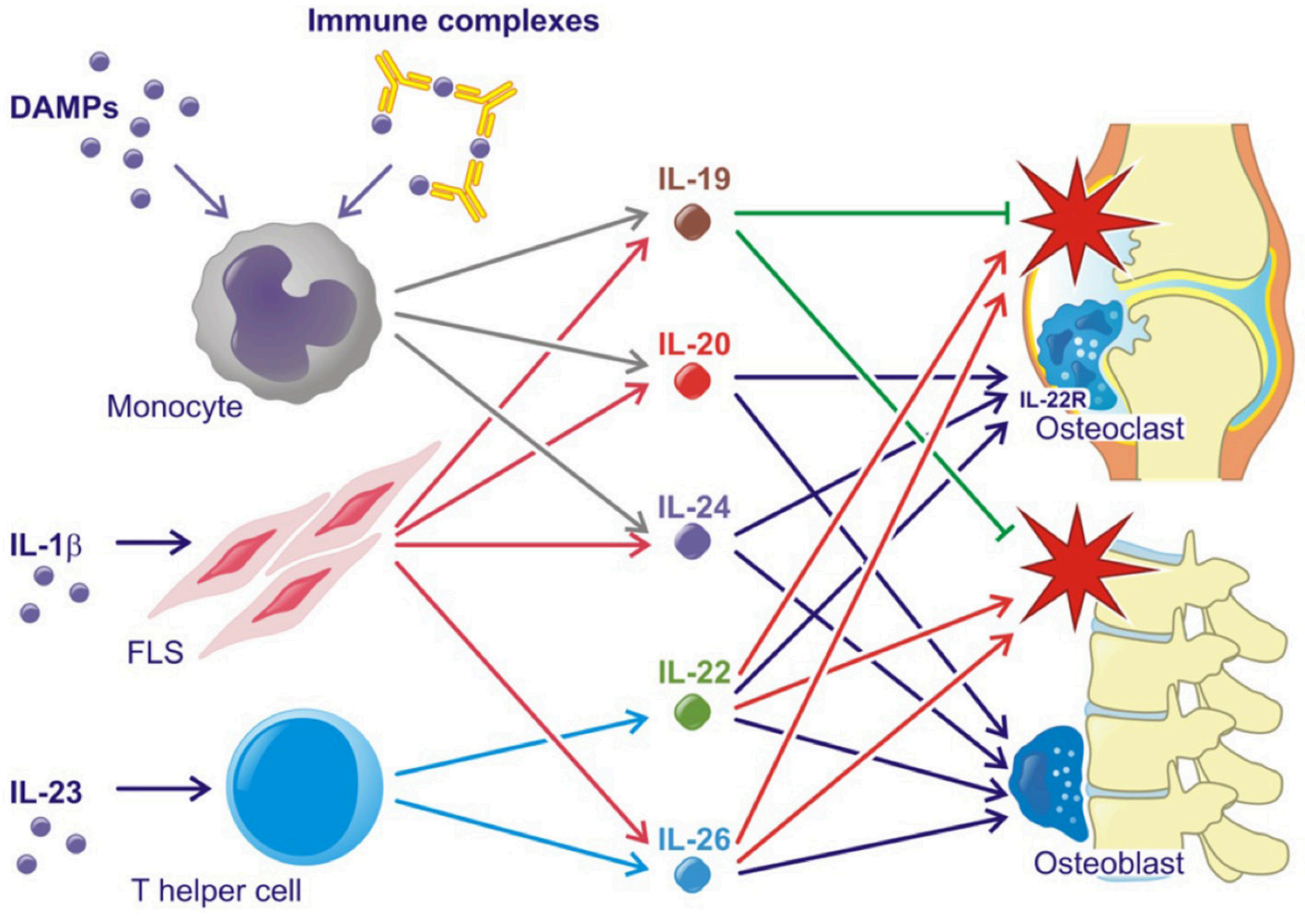
Simplified role of the IL-20 family cytokines in rheumatoid arthritis and spondyloarthritis. Red stars indicate inflammation. Lines with an arrow head indicate stimulation. Lines without arrow head indicate inhibition.

All three IL-20R cytokines are expressed by monocytes, fibroblasts and several epithelial cells. Furthermore, IL-19 and IL-24 are expressed by B cells, IL-20 production can be induced in dendritic cells and IL-24 is expressed by Th2 cells (11, 21, 22). Toll like receptor (TLR)2 and TLR4 agonists and IL-1β increase the production of all three cytokines (23–25) (Figure 2). The induction of the IL-20R cytokines through activation of TLR2 and TLR4 is interesting because several endogenous TLR2 and TLR4 agonists are produced in RA and SpA (e.g., HMGB1 and hyaluronic acid fragments) (26–29). In fact, this pathway may be the primary contributor to the increase in plasma levels of IL-20 and IL-24 in SpA. Myofibroblast pathways are thus activated in SpA, which lead to extracellular matrix turnover and new bone formation (30). In this process, factors from host cells such as fibrinogen, EDA fibronectin, hyaluronic acid, and tenascin C are released. These factors can all function as danger associated molecular patterns (DAMPs) (31). Further, all three cytokines are induced by immune complexes that activate FcγRs in PBMCs. More specifically, citrullinated fibrinogen-containing immune complexes were shown to activate FcγRIIa (Figure 2) (32). The association between the IL-20R cytokines and either IgM-RF and anti-CCP antibodies could be important because these autoantibodies and the generation of immune complexes is an ongoing process even in patients in clinical remission (1). In line with this, the concentration of IL-20 and IL-24 remained increased in some patients with early RA at follow-up after 6 months of treatment compared with HCs in this study (32). Thus, some patients have increased levels of IL-20 and IL-24 even when in clinical remission.

#### Receptor expression

The three receptor subunits are primarily expressed by resident effector cells of target organs (e.g., keratinocytes, osteoclasts, and intestinal and airway epithelial cells) and not on cells traditionally associated with the immune system (21, 22). However, the IL-20RB has been identified on several peripheral blood leukocyte subsets in the expression pattern studies (21, 22), and the IL-20RA and IL-22RA1 can be induced in macrophages in the lung (13). In addition, some studies have reported effects of the IL-20R cytokines on cells of the immune system not known to express the receptor complexes (33–39). These findings have suggested (1) that IL-22RA1 and IL-20RA are only expressed by leukocytes under certain conditions, (2) that the these two receptor subunits are expressed by leukocytes below the detection limit in the expression pattern studies, or (3) that a not yet identified receptor subunit is expressed by leukocytes. In two studies, all three receptor subunits were found on FLSs in the synovial membrane (16, 17). However, in other studies FLSs did not express the receptors and were unresponsive to stimulation with IL-20 and IL-24 (24). Recently, expression of IL-20RA and IL-22RA1 have been shown on peripheral blood and synovial fluid monocyte subsets from RA patients (32). The percentage of monocytes expressing IL-20RA was not different when comparing RA synovial fluid, RA peripheral blood and HC peripheral blood. In contrast, the percentage of monocytes expressing IL-22RA1 was highest in RA synovial fluid and was also increased in RA peripheral blood compared with HC peripheral blood. The percentage of monocytes expressing both IL-20RA and IL-22RA1 was very low in both RA patients and HCs (32).

### Function

The IL-20R axis was first associated with psoriasis (40–46), but later also with other immune-mediated inflammatory diseases such as inflammatory bowel disease (47–51) and arthritis. The IL-20R subunits have now been identified as risk genes for developing an immune-mediated inflammatory disease in three independent genetic studies (52–54).

Most studies have focused on pro-inflammatory roles of IL-20 and IL-24, whereas IL-19 seems to have anti-inflammatory functions. In evidence of this, transgenic mice overexpressing IL-20 (40) and IL-24 (42) develop psoriasis-like skin disease, while IL-19 overexpressing mice have a normal phenotype (13). In contrast, IL-19 knockout mice show increased susceptibility to experimentally induced colitis (47). In line with this, single-nucleotide polymorphisms of the IL-19 gene (but not the IL-20 or IL-24 genes) are protective in psoriasis (41). Likewise, single-nucleotide polymorphisms of the IL-20 and IL-24 genes (but not the IL-19 gene) are associated with juvenile idiopathic arthritis (55, 56). IL-19 has also been suggested to be protective in human colonic inflammation (57), while the role of IL-20 (48, 49) and IL-24 (50, 51) needs further study. Finally, IL-19 has several anti-inflammatory functions in atherosclerotic disease (58). Several studies have found induction of MCP-1 as a pathogenic mechanism of IL-20 and IL-24 function. MCP-1 expression is upregulated in transgenic mice overexpressing IL-24 and IL-20 increases the production of MCP-1 in kidney mesangial cells and intervertebral disc cells (42, 59, 60).

Supporting the tissue homeostasis function of the IL-20R cytokines, the receptor subunits IL-20RA and IL-22RA1 are found on several epithelial cells of target organs such as keratinocytes and lining cells of the gut and airways (13, 21, 22). The two cytokines are upregulated in wound healing and regulate tissue repair mechanisms (61–63). Supporting tissue homestatic properties in arthritis, IL-20 and IL-24 have been shown to increase osteoblast mineralization and are associated with chronic changes in the lumbar spine in SpA (Figure 2) (24). Further, the IL-22RA1 was recently found on osteoclast precursors and osteoclasts. In line with this, IL-20 and IL-24 plasma levels were associated with radiographic progression in early RA patients. Further, IL-20 and IL-24 stimulation led to an increased MCP-1 production in osteoclast cultures (32). MCP-1 is a chemoattractant protein that recruits CCR-2-expressing cells. This receptor is expressed by osteoclast precursors (64–66). Hereby, IL-20 and IL-24 could perpetuate bone destruction by stimulating the recruitment of more monocytes and osteoclast precursors to areas of ongoing bone resorption (Figure 2). These findings are in line with the identified role of IL-20 in osteoporosis (67) and osteoarthritis (68).

### Potential target for disease modification

IL-20 and IL-24 act upon a restricted number of epithelial cell populations and seem to have only moderate effects on leukocytes. Thus, inhibition of the two cytokines may not be associated with increased risk of infection (69). In this way, IL-20 and IL-24 could be potential treatment targets in RA and SpA. The anti-IL-20 drug candidate fletikumab (NN8226, NNC0109-0012) for the treatment of RA has been tested in clinical trials (Table 1). The drug was demonstrated to be safe and well tolerated in the phase 1 trial (NCT01038674) (70). In the Phase 2a trial, neutralization of IL-20 proved effective, especially in seropositive RA patients, which strengthens the link between IL-20 and RF (NCT01282255) (72). However, a Phase 2b trial with this drug candidate very recently failed to reach the primary outcome measure of ACR20 after 12 weeks in RA patients with inadequate responses to either anti-TNFα (NCT01636817) or methotrexate (NCT01636843) therapy. There are several potential reasons why this anti-IL-20 antibody failed in the clinical trial. First, inhibition of IL-20 alone might not be sufficient because of the redundancy in this cytokine family. In line with this, broad blockade of signaling from the IL-20 family of cytokines potently inhibit arthritis in a collagen-induced arthritis (CIA) model (75). Second, the NN8226 antibody (fletikumab) might not have been optimal. However, fletikumab was a human antibody (IgG4) with a high affinity (Kd: 37 pM) and a very high potency in blocking IL-20 mediated activity on IL-20RA/IL-20RB and IL-22RA1/IL20R2 expressing cells (IC50: 0.27 nM).It had a serum half-life of ~3 weeks in RA patients (70). Third, it is possible that the IL-20 axis plays a role only in a small subset of RA patients. Fourth, the primary end point of the clinical trials was disease activity after a short time period. Possibly, IL-20 inhibition could affect other outcomes such as progression of bone erosions after a longer time period. Finally, it could also mean that IL-20 does not play a central role in RA.

**Table 1:** Drugs targeting the IL-20 family of cytokines with relevance to immune mediated inflammatory arthritis.

**Function**	**Name**	**Disease**	**Phase**	**Conclusion**	**Trial and reference**
IL-20 inhibition	Fletikumab (NN8226, NNC0109-0012)	Healthy volunteers, psoriasis, and RA	1	Safe and well tolerated	NCT01038674 (70)
		Psoriasis	1	The expansion phase was terminated early due to apparent lack of efficacy (PASI improvement)	NCT01261767 (71)
		RA	2a	Effect in seropositive RA patients as early as week 1 with further improvements to week 12 (ACR responses)	NCT01282255 (72)
		RA	2b	Ended, no final data released	NCT01636817 and NCT01636843
IL-22 inhibition	Fezakinumab (ILV-094)	Healthy volunteers	1	Ended, no final data released	NCT00434746
		Psoriasis	1	Ended, no final data released	NCT00563524
		RA	2	Ended, no final data released	NCT00883896
		Atopic dermatitis	2	Skin improvement (CONRAD scores)	NCT01941537 (73)
IL-22RA1 inhibition	ARGX-112	Atopic dermatitis	Pre-clinical		
IL-22R activation	Promenakin (F-652)	Colitis	Pre-clinical		
		Acute alcoholic hepatitis	2	Ongoing	NCT02655510
		Graft versus host disease	2	Ongoing	NCT02406651
	RG7880 (UTTR1147A)	Healthy volunteers, ulcerative colitis and Crohn's disease	1	Safe and well tolerated	NCT02749630 (74)
		Diabetic foot ulcer	1	Ongoing	NCT02833389
		Ulcerative colitis	2	Ongoing	NCT03558152
JAK1 or TYK2 inhibition	E.g., tofacitinib and baricitinib	Immune mediated inflammatory diseases	Several	Efficacy in RA, SpA, and psoriatic arthritis	Several published and ongoing studies

Of note, the IL-20R axis signals via the JAK pathway and specifically utilizes the heterodimeric pairing of JAK1 and Tyrosine kinase 2, which in turn activate STAT3. Therefore, it is likely that JAK inhibitors such as tofacitinib (a selective inhibitor of JAK1 and 3) and baricitinib (a selective inhibitor of JAK1 and 2) interupt the intracellular signaling of IL-20R ligands as well as the signaling of other members of the type I/II cytokine family (Table 1) (76). Because the JAK inhibitors are multi-cytokine inhibitors, it is difficult to ascribe the magnitude of efficacy observed in any given immune-mediated inflammatory disorder to signaling via a particular cytokine receptor. However, it should be noted that both baricitinib and tofacitinib have well established efficacy in RA (76) and tofacitinib has demonstrated efficacy in a phase II study of ankylosing spondylitis and in psoriatic arthritis (77, 78).

## IL-22

### Expression and regulation

Another central effector cytokine in the IL-20 family of cytokines is IL-22. Apart from being produced by Th17 cells, IL-22 is also produced by the Th22 cell. Th17 and Th22 cells are closely related, both being CD4+ T helper cells developed under the influence of IL-1β, IL-6, and IL-23 (Figure 2). The development of Th17 and Th22 are dependent on different transcription factors, but they also show differences in their expression of surface receptors. While RAR-related orphan receptor gamma (RORγt) is the transcription factor responsible for Th17 induction, the aryl-hydro-carbon receptor (AHR) together with RORγt are the responsible transcription factors for the development of the Th22 phenotype. Th17 cells are identified through expression of CCR6, CCR4, but not CCR10. By contrast, Th22 cells express CCR4, CCR6 and also CCR10 (79, 80).

IL-22 signals through the heterodimeric receptor complex consisting of the ubiquitously expressed IL-10RB and the more restrictively expressed IL-22RA1 (81). IL-22RA1 is primarily present on epithelial cells and certain monocyte subsets, and very limited IL-22RA1 expression is found on other immune cells (21, 22). IL-22 functions are further regulated by the presence of IL-22 binding protein (BP), a soluble form of the IL-22RA1 (82). IL-22BP is able to bind IL-22, thereby antagonizing the effector functions of IL-22 (83, 84).

### Function

The effect of IL-22 has been described as protective in some settings, where it promotes increased resistance to bacterial infection in the gut, maintains intestinal barrier functions and promotes the production of antimicrobial peptides (85). Further, T cell differentiation toward an IL-22-producing phenotype may also be favorable in alcoholic hepatitis (86). In contrast, in synovial tissue IL-22 is involved in pro-inflammatory reactions such as FLS proliferation and MCP-1 secretion (87).

In animal models, the IL-23-Th17 axis including IL-22 has been associated with several immune-mediated inflammatory conditions. IL-23 double-knockout mice were resistant to the development of CIA and to CNS inflammation in the experimental allergic encephalopathy (EAE) mouse model (88, 89). In line with this, IL-22 production was associated with increased joint destruction in the CIA model (Figure 2) (90). Recently, IL-23 was shown to act on RORγt+ CD3+CD4-CD8- T cells in order to develop SpA-like disease in mice. The mechanisms involved include IL-22 induced genes associated with new bone formation (Figure 2) (91). Animal data further suggest that the IL-22RA1 is the primary driver of disease in the autoinflammatory phenotype. Transgenic mice overexpressing IL-22 develop psoriasis-like disease, which is inhibited after breeding these mice with IL-22RA1 knockout mice (but not with IL-20RA knockout mice) (92). Furthermore, IL-22RA1 transgenic mice develop a systemic inflammatory condition. Eliminating the effect of all three cytokines in the IL-20RB knockout mice attenuates disease in a mouse psoriasis model (93), but upregulates antigen-specific T cell responses (94).

In humans, IL-22 has also been associated with diseases involving the IL-23-Th17 axis such as SpA including psoriatic arthritis (95). Adding to a potential role in these diseases characterized by new bone formation, IL-22 stimulates mineralization by ligament cells in odontologic disease (96). IL-22 has further been shown to be upregulated in RA patients and associated with radiographic progression and disease activity (97, 98).

### Potential target for disease modification

Two studies of the IL-22 inhibitor fezakinumab (ILV-094) in psoriasis (NCT00563524) and RA (NCT00883896) were completed several years ago with no final data released (Table 1). This indicates that IL-22 might not have a central role in immune-mediated inflammatory arthritis. Very recently, fezakinumab showed clinical efficacy in atopic dermatitis (73). ZymoGenetics developed an IL-22RA1 antibody but the development was discontinued. More recently, ArGenX in collaboration with Leo Pharma have developed an IL-22RA1 antibody (ARGX-112) and plan clinical testing in atopic dermatitis (Table 1). Roche together with Genentech (RG7880, UTTR1147A) and Generon (Promenakin, F-652) develop IL-22 agonists for the treatment of ulcerative colitis with ongoing trials also in pancreatitis, acute alcoholic hepatitis, graft versus host disease and treatment of diabetic foot ulcers (Table 1). Treatment with these recombinant IL-22 fusion proteins seems to be safe (74, 99). No studies that interfere with the IL-22 receptor have been conducted in immune mediated inflammatory arthritis. However, modulation of IL-22 signaling by JAK inhibition could contribute to the clinical effect seen with these treatments (76, 100, 101).

## IL-26

### Expression and regulation

IL-26, also denominated AK155, is a highly cationic charged cytokine initially found in activated T-cells and NK cells as part of the IL-10 cytokine family, and later classified as a Th17 cytokine (102). Recent studies have revealed IL-26 expression in a number of other cell types, including alveolar macrophages (103), bronchial epithelial cells (104), synoviolin+ synovial cells from RA patients (36), and myofibroblasts of spondyloarthritis patients (105) suggesting that IL-26 production occurs in multiple cell types. There is no functional mouse IL-26 gene, which complicates the characterization of this cytokine in preclinical settings.

The IL-26 receptor is a heterodimer composed of IL-10RB and IL-20RA. The IL-10RB monomer is ubiquitously expressed, and shares with IL-10, IL-22, and interferons, while the more sparsely expressed IL-20RA monomer is shared with IL-19, IL-20, and IL-24 (106), and constitutes the limiting factor of the cells ability to respond to IL-26 (106, 107). Expression of IL-20RA is upregulated in psoriatic skin (40), but also reported present on osteoclasts and osteoblasts (108).

### Function

The effector functions of IL-26 are still poorly defined. However, it seems that IL-26 primarily act as part of the anti-microbial response against bacteria by recruiting neutrophils to the site of infection (103). When IL-26 forms complexes with extracellular DNA, bacterial or self-DNA (109, 110), the IL-26-DNA complexes can be transported into the cytosol of myeloid cells or trigger type 1 interferon production by plasmacytoid dendritic cells and activate monocytes via STimulator of INterferon Genes (STING) and the inflammasome (109, 110). Elevated levels of IL-26 and IL-26-DNA complexes are seen in a variety of active inflammatory diseases (105).

In immune mediated inflammatory diseases, IL-26 is suggested to be both an important driver of Th17 differentiation and a key Th17 effector cytokine (Figure 2). Thus, IL-26 stimulated monocytes are reported to promote Th-17 cell generation from non-Th17-committed memory T-cells in RA (36), However, IL-26 is also produced by Th17 cells in colonic lesions of patients with active inflammatory bowel disease (IBD) (111).

In RA, IL-26 acts as a pro-inflammatory cytokine, which is constitutively expressed by synovial cells and induce cytokine secretion by myeloid cells leading to Th17 cell generation (Figure 2). Single-nucleotide polymorphisms (SNPs) associated with RA, as well as other inflammatory disorders, like IBD, have been identified in the IL26 gene (112–114).

In SpA, levels of IL-26 are elevated in synovial fluid compared with plasma which suggests that IL-26 is produced locally in the inflamed joint. IL-26 was primarily produced by α-smooth-muscle-actin expressing fibroblasts, called myofibroblasts. This subtype of fibroblast is specifically upregulated in SpA (115) and is a contributor to fibrosis in several immune mediated diseases (116). Osteoblasts treated with IL-26 show increased mineralization and IL-26 could therefore play a pro-fibrotic role in SpA as well as induce inflammation (Figure 2). Supporting this association, a fibrotic effect of IL-26 was also reported in graft versus host disease (117).

### Potential target for disease modification

The role of IL-26 in the innate immune defense against bacterial infections complicates the use of the cytokine as a therapeutic target of chronic immune mediated inflammatory diseases. However, the fibrotic properties of the cytokine makes the cytokine interesting as a therapeutic target. Thus, TNFα and IL-17A inhibitors mostly target inflammation while only slowing down tendon calcification at a moderate level.

No medical therapeutics have currently been developed against IL-26. This might partly be due to the lack of IL-26 in mice and rats which complicates the initial pharmaceutical experiments (118). IL-26 signals through JAK1 and Tyrosine kinase 2 and STAT1 and 3 (106, 119). IL-26 is therefore also inhibited by drugs targeting the JAK molecules, e.g., tofacitinib and baricitinib (Table 1) (76).

## Conclusion

IL-19 seems to have an anti-inflammatory role in arthritis. IL-20 and IL-24 have been associated with bone degradation and radiographic progression. Potentiation of MCP-1 from osteoclasts could be a mechanism contributing to this, but other effects of IL-20 and IL-24 on osteoclasts cannot be excluded. IL-22 has been associated with progression of bone erosion and IL-26 was shown to induce several proinflammatory cytokines in RA. The strategy for modulation should take into account the effects of all IL-20 family members. This redundancy encourages inhibition of more than one cytokine or one of the shared receptors in this cytokine family. However, the roles of this cytokine family could be limited in human immune-mediated arthritis or only relevant in a subset of patients or for certain aspects of the diseases. All of the IL-20 family members signal through the JAK-STAT pathway, and therefore are potential targets for drugs that inhibit this pathway. Effects and adverse effects in ongoing clinical trials with inhibitors of IL-22 and the IL-22RA1 subunit and recombinant IL-22 fusion proteins will possibly give important information about the IL-20 subfamily of cytokines in the future.

## Author contributions

TK, TA, and LH drafted the manuscript. MH, JG, PS, PT, LS, and BD made substantial contributions to the first draft. TK finalized the manuscript. All authors read and approved the final manuscript.

### Conflict of interest statement

JG and PS are former employees in Novo Nordisk working on the anti-IL-20 drug candidate fletikumab. LS was principal investigator on the phase 2a study of the anti-IL-20 drug candidate fletikumab for the treatment of RA. JG is now employed by Eli Lilly. PS is now employed by Celgene. The remaining authors declare that the research was conducted in the absence of any commercial or financial relationships that could be construed as a potential conflict of interest.

## References

[1] Scott DL Wolfe F Huizinga TWJ. Rheumatoid arthritis. Lancet (2010) 376:1094–108. 10.1016/S0140-6736(10)60826-4 20870100

[2] Dougados M Baeten D. Spondyloarthritis. Lancet (2011) 377:2127–37. 10.1016/S0140-6736(11)60071-8 21684383

[3] Lories RJ Baeten DLP. Differences in pathophysiology between rheumatoid arthritis and ankylosing spondylitis. Clin Exp Rheumatol. (2009) 27:S10–4. 19822039

[4] Schett G Gravallese E. Bone erosion in rheumatoid arthritis: mechanisms, diagnosis and treatment. Nat Rev Rheumatol. (2012) 8:656–64. 10.1038/nrrheum.2012.153 23007741 PMC4096779

[5] Goldring SR. Osteoimmunology and bone homeostasis: relevance to spondyloarthritis. Curr Rheumatol Rep. (2013) 15:342–6. 10.1007/s11926-013-0342-2 23709258 PMC3689465

[6] Will R Palmer R Bhalla AK Ring F Calin A. Osteoporosis in early ankylosing spondylitis: a primary pathological event? Lancet (1989) 2:1483–5. 2574769 10.1016/s0140-6736(89)92932-2

[7] Burmester GR Feist E Dörner T. Emerging cell and cytokine targets in rheumatoid arthritis. Nat Rev Rheumatol. (2014) 10:77–88. 10.1038/nrrheum.2013.168 24217582

[8] Taylor PC. Developing anti-TNF and biologic agents. Rheumatology (2011) 50:1351–3. 10.1093/rheumatology/ker235 21746890

[9] Choy EH Kavanaugh AF Jones SA. The problem of choice: current biologic agents and future prospects in RA. Nat Rev Rheumatol. (2013) 9:154–63. 10.1038/nrrheum.2013.8 23419427

[10] Hofmann SR Rösen-Wolff A Tsokos GC Hedrich CM. Biological properties and regulation of IL-10 related cytokines and their contribution to autoimmune disease and tissue injury. Clin Immunol. (2012) 143:116–27. 10.1016/j.clim.2012.02.005 22459704

[11] Rutz S Wang X Ouyang W. The IL-20 subfamily of cytokines - from host defence to tissue homeostasis. Nat Rev Immunol. (2014) 14:783–95. 10.1038/nri3766 25421700

[12] Dumoutier L Leemans C Lejeune D Kotenko SV Renauld JC. Cutting edge: STAT activation by IL-19, IL-20 and mda-7 through IL-20 receptor complexes of two types. J Immunol. (2001) 167:3545–9. 10.4049/jimmunol.167.7.3545 11564763

[13] Parrish-Novak J Xu W Brender T Yao L Jones C West J. Interleukins 19, 20, and 24 signal through two distinct receptor complexes. Differences in receptor-ligand interactions mediate unique biological functions. J Biol Chem. (2002) 277:47517–23. 10.1074/jbc.M205114200 12351624

[14] Logsdon NJ Deshpande A Harris BD Rajashankar KR Walter MR. Structural basis for receptor sharing and activation by interleukin-20 receptor-2 (IL-20R2) binding cytokines. Proc Natl Acad Sci USA. (2012) 109:12704–9. 10.1073/pnas.1117551109 22802649 PMC3412030

[15] Kragstrup TW Otkjaer K Holm C Jørgensen A Hokland M Iversen L. The expression of IL-20 and IL-24 and their shared receptors are increased in rheumatoid arthritis and spondyloarthropathy. Cytokine (2008) 41:16–23. 10.1016/j.cyto.2007.10.004 18061474

[16] Hsu Y-H Li H-H Hsieh M-Y Liu M-F Huang K-Y Chin L-S. Function of interleukin-20 as a proinflammatory molecule in rheumatoid and experimental arthritis. Arthritis Rheum. (2006) 54:2722–33. 10.1002/art.22039 16947773

[17] Sakurai N Kuroiwa T Ikeuchi H Hiramatsu N Maeshima A Kaneko Y. Expression of IL-19 and its receptors in RA: potential role for synovial hyperplasia formation. Rheumatology (2008) 47:815–20. 10.1093/rheumatology/ken061 18397956

[18] Šenolt L Prajzlerová K Hulejová H Šumová B Filková M Veigl D. Interleukin-20 is triggered by TLR ligands and associates with disease activity in patients with rheumatoid arthritis. Cytokine (2017) 97:187–92. 10.1016/j.cyto.2017.06.009 28662439

[19] Scrivo R Conigliaro P Riccieri V Di Franco M Alessandri C Spadaro A. Distribution of interleukin-10 family cytokines in serum and synovial fluid of patients with inflammatory arthritis reveals different contribution to systemic and joint inflammation. Clin Exp Immunol. (2015) 179:300–8. 10.1111/cei.12449 25178435 PMC4298407

[20] Valentina M Jan F Peder NL Bo Z Hongjie D Pernille K. Cytokine detection and simultaneous assessment of rheumatoid factor interference in human serum and synovial fluid using high-sensitivity protein arrays on plasmonic gold chips. BMC Biotechnol. (2015) 15:73. 10.1186/s12896-015-0186-0 26268325 PMC4535377

[21] Wolk K Kunz S Asadullah K Sabat R. Cutting edge: immune cells as sources and targets of the IL-10 family members? J Immunol. (2002) 168:5397–402. 10.4049/jimmunol.168.11.5397 12023331

[22] Nagalakshmi ML Murphy E McClanahan T de Waal Malefyt R. Expression patterns of IL-10 ligand and receptor gene families provide leads for biological characterization. Int Immunopharmacol. (2004) 4:577–92. 10.1016/j.intimp.2004.01.007 15120644

[23] Kragstrup TW Andersen T Holm C Schiøttz-Christensen B Jurik AG Hvid M. Toll-like receptor 2 and 4 induced interleukin-19 dampens immune reactions and associates inversely with spondyloarthritis disease activity. Clin Exp Immunol. (2015) 180:233–42. 10.1111/cei.12577 25639337 PMC4408158

[24] Kragstrup TW Andersen MN Schiøttz-Christensen B Jurik AG Hvid M Deleuran B. Increased interleukin (IL)-20 and IL-24 target osteoblasts and synovial monocytes in spondyloarthritis. Clin Exp Immunol. (2017) 189:342–51. 10.1111/cei.12973 28369789 PMC5543495

[25] Alanärä T Karstila K Moilanen T Silvennoinen O Isomäki P. Expression of IL-10 family cytokines in rheumatoid arthritis: elevated levels of IL-19 in the joints. Scand J Rheumatol. (2010) 39:118–26. 10.3109/03009740903170823 20001767

[26] Oktayoglu P Em S Tahtasiz M Bozkurt M Ucar D Yazmalar L. Elevated serum levels of high mobility group box protein 1 (HMGB1) in patients with ankylosing spondylitis and its association with disease activity and quality of life. Rheumatol Int. (2013) 33:1327–31. 10.1007/s00296-012-2578-y 23143556

[27] Duruöz MT Turan Y Cerrahoglu L Isbilen B. Serum hyaluronic acid levels in patients with ankylosing spondylitis. Clin Rheumatol. (2008) 27:621–6. 10.1007/s10067-007-0757-0 17955278

[28] Goh FG Midwood KS. Intrinsic danger: activation of Toll-like receptors in rheumatoid arthritis. Rheumatology (2012) 51:7–23. 10.1093/rheumatology/ker257 21984766

[29] Huang Q-Q Pope RM. The role of Toll-like receptors in rheumatoid arthritis. Curr Rheumatol Rep. (2009) 11:357–64. 10.1007/s11926-009-0051-z 19772831 PMC2913446

[30] Beyer C Distler JHW. Changing paradigms in spondylarthritis: the myofibroblast signature. Arthritis Rheum. (2012) 65:24–7. 10.1002/art.37703 22972392

[31] Chen GY Nuñez G. Sterile inflammation: sensing and reacting to damage. Nat Rev Immunol. (2010) 10:826–37. 10.1038/nri2873 21088683 PMC3114424

[32] Kragstrup TW Greisen SR Nielsen MA Rhodes C Stengaard-Pedersen K Hetland ML. The interleukin-20 receptor axis in early rheumatoid arthritis: novel links between disease-associated autoantibodies and radiographic progression. Arthritis Res Ther. (2016) 18:61. 10.1186/s13075-016-0964-7 26968800 PMC4788924

[33] Liao S-C Cheng Y-C Wang Y-C Wang C-W Yang S-M Yu C-K. IL-19 induced Th2 cytokines and was up-regulated in asthma patients. J Immunol. (2004) 173:6712–8. 10.4049/jimmunol.173.11.6712 15557163

[34] Jordan WJ Eskdale J Boniotto M Lennon GP Peat J Campbell JDM. Human IL-19 regulates immunity through auto-induction of IL-19 and production of IL-10. Eur J Immunol. (2005) 35:1576–82. 10.1002/eji.200425317 15827959

[35] Oral HB Kotenko SV Yilmaz M Mani O Zumkehr J Blaser K. Regulation of T cells and cytokines by the interleukin-10 (IL-10)-family cytokines IL-19, IL-20, IL-22, IL-24 and IL-26. Eur J Immunol. (2006) 36:380–8. 10.1002/eji.200425523 16365913

[36] Corvaisier M Delneste Y Jeanvoine H Preisser L Blanchard S Garo E. IL-26 is overexpressed in rheumatoid arthritis and induces proinflammatory cytokine production and Th17 cell generation. PLoS Biol. (2012) 10:e1001395. 10.1371/journal.pbio.1001395 23055831 PMC3463509

[37] Hoffman C Park S-H Daley E Emson C Louten J Sisco M. Interleukin-19: a constituent of the regulome that controls antigen presenting cells in the lungs and airway responses to microbial products. PLoS ONE (2011) 6:e27629. 10.1371/journal.pone.0027629 22110701 PMC3217014

[38] Gallagher G Eskdale J Jordan W Peat J Campbell J Boniotto M. Human interleukin-19 and its receptor: a potential role in the induction of Th2 responses. Int Immunopharmacol. (2004) 4:615–26. 10.1016/j.intimp.2004.01.005 15120647

[39] Buzas K Oppenheim JJ Zack Howard OM. Myeloid cells migrate in response to IL-24. Cytokine (2011) 55:429–34. 10.1016/j.cyto.2011.05.018 21703864 PMC3148305

[40] Blumberg H Conklin D Xu WF Grossmann A Brender T Carollo S. Interleukin 20: discovery, receptor identification, and role in epidermal function. Cell (2001) 104:9–19. 10.1016/S0092-8674(01)00187-8 11163236

[41] Kõks S Kingo K Rätsep R Karelson M Silm H Vasar E. Combined haplotype analysis of the interleukin-19 and−20 genes: relationship to plaque-type psoriasis. Genes Immun. (2004) 5:662–7. 10.1038/sj.gene.6364141 15496954

[42] He M Liang P. IL-24 transgenic mice: in vivo evidence of overlapping functions for IL-20, IL-22, and IL-24 in the epidermis. J Immunol. (2010) 184:1793–8. 10.4049/jimmunol.0901829 20061404

[43] Wei C-C Chen W-Y Wang Y-C Chen P-J Lee JY-Y Wong T-W. Detection of IL-20 and its receptors on psoriatic skin. Clin Immunol. (2005) 117:65–72. 10.1016/j.clim.2005.06.012 16043414

[44] Otkjaer K Kragballe K Johansen C Funding AT Just H Jensen UB. IL-20 gene expression is induced by IL-1beta through mitogen-activated protein kinase and NF-kappaB-dependent mechanisms. J Invest Dermatol. (2007) 127:1326–36. 10.1038/sj.jid.5700713 17255956

[45] Otkjaer K Kragballe K Funding AT Clausen JT Noerby PL Steiniche T. The dynamics of gene expression of interleukin-19 and interleukin-20 and their receptors in psoriasis. Br J Dermatol. (2005) 153:911–8. 10.1111/j.1365-2133.2005.06800.x 16225599

[46] Kunz S Wolk K Witte E Witte K Doecke W-D Volk H-D. Interleukin (IL)-19, IL-20 and IL-24 are produced by and act on keratinocytes and are distinct from classical ILs. Exp Dermatol. (2006) 15:991–1004. 10.1111/j.1600-0625.2006.00516.x 17083366

[47] Azuma Y-T Matsuo Y Kuwamura M Yancopoulos GD Valenzuela DM Murphy AJ. Interleukin-19 protects mice from innate-mediated colonic inflammation. Inflamm Bowel Dis. (2010) 16:1017–28. 10.1002/ibd.21151 19834971

[48] Fonseca-Camarillo G Furuzawa-Carballeda J Llorente L Yamamoto-Furusho JK. IL-10– and IL-20–expressing epithelial and inflammatory cells are increased in patients with ulcerative colitis. J Clin Immunol. (2013) 33:640–8. 10.1007/s10875-012-9843-4 23207823

[49] Yamamoto-Furusho JK De-León-Rendón JL la Torre de MG Alvarez-León E Vargas-Alarcón G. Genetic polymorphisms of interleukin 20 (IL-20) in patients with ulcerative colitis. Immunol Lett. (2012) 149:50–3. 10.1016/j.imlet.2012.11.008 23183096

[50] Camarillo GF Furuzawa-Carballeda J Martínez-Benítez B Barreto-Zúñiga R Yamamoto-Furusho JK. Role of the interleukin 24 in patients with ulcerative colitis. Inflamm Bowel Dis. (2011) 17:2209–10. 10.1002/ibd.21635 21287675

[51] Andoh A Shioya M Nishida A Bamba S Tsujikawa T Kim-Mitsuyama S. Expression of IL-24, an activator of the JAK1/STAT3/SOCS3 cascade, is enhanced in inflammatory bowel disease. J Immunol. (2009) 183:687–95. 10.4049/jimmunol.0804169 19535621

[52] McGovern A Schoenfelder S Martin P Massey J Duffus K Plant D. Capture Hi-C identifies a novel causal gene, IL20RA, in the pan-autoimmune genetic susceptibility region 6q23. Genome Biol. (2016) 17:212. 10.1186/s13059-016-1078-x 27799070 PMC5088679

[53] Wu J Yang S Yu D Gao W Liu X Zhang K CRISPR/cas9 mediated knockout of an intergenic variant rs6927172 identified IL-20RA as a new risk gene for multiple autoimmune diseases. Genes Immun. (2018) 17:160 10.1038/s41435-018-0011-6 29483615

[54] Okada Y Wu D Trynka G Raj T Terao C Ikari K. Genetics of rheumatoid arthritis contributes to biology and drug discovery. Nature (2014) 506:376–81. 10.1038/nature12873 24390342 PMC3944098

[55] Omoyinmi E Forabosco P Hamaoui R Bryant A Hinks A Ursu S. Association of the IL-10 gene family locus on chromosome 1 with Juvenile Idiopathic Arthritis (JIA). PLoS ONE (2012) 7:e47673. 10.1371/journal.pone.0047673 23094074 PMC3475696

[56] Fife MS Gutierrez A Ogilvie EM Stock CJW Samuel JM Thomson W. Novel IL10 gene family associations with systemic juvenile idiopathic arthritis. Arthritis Res Ther. (2006) 8:R148. 10.1186/ar2041 16959027 PMC1779449

[57] Yamamoto-Furusho JK Alvarez-León E Fragoso JM Gozalishvilli A Vallejo M Vargas-Alarcón G. Protective role of interleukin-19 gene polymorphisms in patients with ulcerative colitis. Hum Immunol. (2011) 72:1029–32. 10.1016/j.humimm.2011.08.013 21925224

[58] England RN Autieri MV. Anti-inflammatory effects of interleukin-19 in vascular disease. Int J Inflam. (2012) 2012:253583. 10.1155/2012/253583 22844641 PMC3403192

[59] Li H-H Cheng H-H Sun K-H Wei C-C Li C-F Chen W-C. Interleukin-20 targets renal mesangial cells and is associated with lupus nephritis. Clin Immunol. (2008) 129:277–85. 10.1016/j.clim.2008.07.006 18771958

[60] Huang K-Y Lin R-M Chen W-Y Lee C-L Yan J-J Chang M-S. IL-20 may contribute to the pathogenesis of human intervertebral disc herniation. Spine (2008) 33:2034–40. 10.1097/BRS.0b013e31817eb872 18758357

[61] Poindexter NJ Williams RR Powis G Jen E Caudle AS Chada S. IL-24 is expressed during wound repair and inhibits TGFalpha-induced migration and proliferation of keratinocytes. Exp Dermatol. (2010) 19:714–22. 10.1111/j.1600-0625.2010.01077.x 20545760 PMC3161412

[62] Bosanquet DC Harding KG Ruge F Sanders AJ Jiang WG. Expression of IL-24 and IL-24 receptors in human wound tissues and the biological implications of IL-24 on keratinocytes. Wound Repair Regen. (2012) 20:896–903. 10.1111/j.1524-475X.2012.00840.x 23110359

[63] Sa SM Valdez PA Wu J Jung K Zhong F Hall L. The effects of IL-20 subfamily cytokines on reconstituted human epidermis suggest potential roles in cutaneous innate defense and pathogenic adaptive immunity in psoriasis. J Immunol. (2007) 178:2229–40. 10.4049/jimmunol.178.4.2229 17277128

[64] Komano Y Nanki T Hayashida K Taniguchi K Miyasaka N. Identification of a human peripheral blood monocyte subset that differentiates into osteoclasts. Arthritis Res Ther. (2006) 8:R152. 10.1186/ar2046 16987426 PMC1779441

[65] Geissmann F Jung S Littman DR. Blood monocytes consist of two principal subsets with distinct migratory properties. Immunity (2003) 19:71–82. 10.1016/S1074-7613(03)00174-2 12871640

[66] Matsubara R Kukita T Ichigi Y Takigawa I Qu P-F Funakubo N Miyamoto H Nonaka K Kukita A. Characterization and identification of subpopulations of mononuclear preosteoclasts induced by TNF-α in combination with TGF-β in rats. PLoS ONE (2012) 7:e47930. 10.1371/journal.pone.0047930 23110133 PMC3480460

[67] Hsu Y-H Chen W-Y Chan C-H Wu C-H Sun Z-J Chang M-S. Anti-IL-20 monoclonal antibody inhibits the differentiation of osteoclasts and protects against osteoporotic bone loss. J Exp Med. (2011) 208:1849–61. 10.1084/jem.20102234 21844205 PMC3171097

[68] Hsu Y-H Yang Y-Y Huwang M-H Weng Y-H Jou I-M Wu P-T. Anti-IL-20 monoclonal antibody inhibited inflammation and protected against cartilage destruction in murine models of osteoarthritis. PLoS ONE (2017) 12:e0175802. 10.1371/journal.pone.0175802 28426699 PMC5398531

[69] Leng R-X Pan H-F Tao J-H Ye D-Q. IL-19, IL-20 and IL-24: potential therapeutic targets for autoimmune diseases. Expert Opin Ther Targets (2011) 15:119–26. 10.1517/14728222.2011.534461 21073280

[70] Lundblad MS Overgaard RV Göthberg M Fjording MS Watson E Clinical pharmacokinetics of the anti-interleukin-20 monoclonal antibody NNC0109-0012 in healthy volunteers and patients with psoriasis or rheumatoid arthritis. Adv Ther. (2015) 1–11. 10.1007/s12325-015-0191-7 25749867

[71] Gottlieb AB Krueger JG Sandberg Lundblad M Göthberg M Skolnick BE. First-in-human, phase 1, randomized, dose-escalation trial with recombinant anti-IL-20 monoclonal antibody in patients with psoriasis. PLoS ONE (2015) 10:e0134703. 10.1371/journal.pone.0134703 26252485 PMC4529098

[72] Šenolt L Leszczynski P Dokoupilová E Göthberg M Valencia X Hansen BB. Efficacy and Safety of Anti-Interleukin-20 Monoclonal Antibody in Patients With Rheumatoid Arthritis: A Randomized Phase IIa Trial. Arthrit Rheumatol. (2015) 67:1438–48. 10.1002/art.39083 25707477

[73] Guttman-Yassky E Brunner PM Neumann AU Khattri S Pavel AB Malik K. Efficacy and safety of fezakinumab (an IL-22 monoclonal antibody) in adults with moderate-to-severe atopic dermatitis inadequately controlled by conventional treatments: a randomized, double-blind, phase 2a trial. J Am Acad Dermatol. (2018) 78:872–881.e6. 10.1016/j.jaad.2018.01.016 29353025 PMC8711034

[74] Rothenberg ME Wang Y Lekkerkerker A Danilenko DM Maciuca R Erickson R Randomized phase I healthy volunteer study of UTTR1147A (IL-22Fc): a potential therapy for epithelial injury. Clin Pharmacol Ther. (2018) 33:747 10.1002/cpt.1164 29952004

[75] Liu X Zhou H Huang X Cui J Long T Xu Y. A broad blockade of signaling from the IL-20 family of cytokines potently attenuates collagen-induced arthritis. J Immunol. (2016) 197:3029–37. 10.4049/jimmunol.1600399 27619991

[76] O'Shea JJ Schwartz DM Villarino AV Gadina M McInnes IB Laurence A. The JAK-STAT pathway: impact on human disease and therapeutic intervention. Annu Rev Med. (2015) 66:311–28. 10.1146/annurev-med-051113-024537 25587654 PMC5634336

[77] van der Heijde D Deodhar A Wei JC Drescher E Fleishaker D Hendrikx T. Tofacitinib in patients with ankylosing spondylitis: a phase II, 16-week, randomised, placebo-controlled, dose-ranging study. Ann Rheum Dis. (2017) 76:1340–7. 10.1136/annrheumdis-2016-210322 28130206 PMC5738601

[78] Mease P Hall S FitzGerald O van der Heijde D Merola JF Avila-Zapata F Tofacitinib or adalimumab versus placebo for psoriatic arthritis. N Engl J Med. (2017) 377:1537–50. 10.1056/NEJMoa1615975 29045212

[79] Duhen T Geiger R Jarrossay D Lanzavecchia A Sallusto F Production of interleukin 22 but not interleukin 17 by a subset of human skin-homing memory T cells. Nat Immunol. (2009) 10:857–63. 10.1038/ni.1767 19578369

[80] Trifari S Kaplan CD Tran EH Crellin NK Spits H Identification of a human helper T cell population that has abundant production of interleukin 22 and is distinct from TH-17, TH1 and TH2 cells. Nat Immunol. (2009) 10:864–71. 10.1038/ni.1770 19578368

[81] Kotenko SV Izotova LS Mirochnitchenko OV Esterova E Dickensheets H Donnelly RP. Identification of the functional interleukin-22 (IL-22) receptor complex: the IL-10R2 chain (IL-10Rbeta) is a common chain of both the IL-10 and IL-22 (IL-10-related T cell-derived inducible factor, IL-TIF) receptor complexes. J Biol Chem. (2001) 276:2725–32. 10.1074/jbc.M007837200 11035029

[82] Kotenko SV Izotova LS Mirochnitchenko OV Esterova E Dickensheets H Donnelly RP. Identification, cloning, and characterization of a novel soluble receptor that binds IL-22 and neutralizes its activity. J Immunol. (2001) 166:7096–103. 10.4049/jimmunol.166.12.7096 11390454

[83] Jones BC Logsdon NJ Walter MR. Structure of IL-22 bound to its high-affinity IL-22R1 chain. Structure (2008) 16:1333–44. 10.1016/j.str.2008.06.005 18599299 PMC2637415

[84] Dumoutier L Lejeune D Colau D Renauld JC. Cloning and characterization of IL-22 binding protein, a natural antagonist of IL-10-related T cell-derived inducible factor/IL-22. J Immunol. (2001) 166:7090–5. 10.4049/jimmunol.166.12.7090 11390453

[85] Liang SC Tan X-Y Luxenberg DP Karim R Dunussi-Joannopoulos K. Interleukin (IL)-22 and IL-17 are coexpressed by Th17 cells and cooperatively enhance expression of antimicrobial peptides. J Exp Med. (2006) 203:2271–9. 10.1084/jem.20061308 16982811 PMC2118116

[86] Støy S Sandahl TD Dige AK Agnholt J Rasmussen TK Grønbæk H. Highest frequencies of interleukin-22-producing T helper cells in alcoholic hepatitis patients with a favourable short-term course. PLoS ONE (2013) 8:e55101. 10.1371/journal.pone.0055101 23372820 PMC3555927

[87] Ikeuchi H Kuroiwa T Hiramatsu N Kaneko Y Hiromura K Ueki K. Expression of interleukin-22 in rheumatoid arthritis: potential role as a proinflammatory cytokine. Arthritis Rheum. (2005) 52:1037–46. 10.1002/art.20965 15818686

[88] Cua DJ Sherlock J Chen Y Murphy CA Joyce B Seymour B. Interleukin-23 rather than interleukin-12 is the critical cytokine for autoimmune inflammation of the brain. Nature (2003) 421:744–8. 10.1038/nature01355 12610626

[89] Langrish CL Chen Y Blumenschein WM Mattson J Basham B Sedgwick JD. IL-23 drives a pathogenic T cell population that induces autoimmune inflammation. J Exp Med. (2005) 201:233–40. 10.1084/jem.20041257 15657292 PMC2212798

[90] Marijnissen RJ Koenders MI Smeets RL Stappers MHT Nickerson Nutter C Joosten LAB. Increased expression of interleukin-22 by synovial Th17 cells during late stages of murine experimental arthritis is controlled by interleukin-1 and enhances bone degradation. Arthritis Rheum. (2011) 63:2939–48. 10.1002/art.30469 21618207

[91] Sherlock JP Joyce-Shaikh B Turner SP Chao C-C Sathe M Grein J. IL-23 induces spondyloarthropathy by acting on ROR-γt+ CD3+CD4–CD8– entheseal resident T cells. Nat Med. (2012) 18:1069–76. 10.1038/nm.2817 22772566

[92] Wolk K Haugen HS Xu W Witte E Waggie K Anderson M. IL-22 and IL-20 are key mediators of the epidermal alterations in psoriasis while IL-17 and IFN-gamma are not. J Mol Med. (2009) 87:523–36. 10.1007/s00109-009-0457-0 19330474

[93] Chan JR Blumenschein W Murphy E Diveu C Wiekowski M Abbondanzo S. IL-23 stimulates epidermal hyperplasia via TNF and IL-20R2-dependent mechanisms with implications for psoriasis pathogenesis. J Exp Med. (2006) 203:2577–87. 10.1084/jem.20060244 17074928 PMC2118145

[94] Wahl C Müller W Leithäuser F Adler G Oswald F Reimann J. IL-20 receptor 2 signaling down-regulates antigen-specific T cell responses. J Immunol. (2009) 182:802–10. 10.4049/jimmunol.182.2.802 19124723

[95] Mitra A Raychaudhuri SK Raychaudhuri SP. Functional role of IL-22 in psoriatic arthritis. Arthritis Res Ther. (2012) 14:R65. 10.1186/ar3781 22417743 PMC3446433

[96] Kato-Kogoe N Nishioka T Kawabe M Kataoka F Yamanegi K Yamada N. The promotional effect of IL-22 on mineralization activity of periodontal ligament cells. Cytokine (2012) 59:41–8. 10.1016/j.cyto.2012.03.024 22537848

[97] da Rocha LF Duarte ÂLBP Dantas AT Mariz HA Pitta IDR Galdino SL. Increased serum interleukin 22 in patients with rheumatoid arthritis and correlation with disease activity. J Rheumatol. (2012) 39:1320–5. 10.3899/jrheum.111027 22589261

[98] Leipe J Schramm MA Grunke M Baeuerle M Dechant C Nigg AP. Interleukin 22 serum levels are associated with radiographic progression in rheumatoid arthritis. Ann Rheum Dis. (2011) 70:1453–7. 10.1136/ard.2011.152074 21593004

[99] Tang K-Y Lickliter J Huang Z-H Xian Z-S Chen H-Y Huang C Safety, pharmacokinetics, and biomarkers of F-652, a recombinant human interleukin-22 dimer, in healthy subjects. Cell Mol Immunol. (2018) 13:21 10.1038/s41423-018-0029-8 PMC6474205 29670279

[100] Sabat R Ouyang W Wolk K. Therapeutic opportunities of the IL-22-IL-22R1 system. Nat Rev Drug Discov. (2014) 13:21–38. 10.1038/nrd4176 24378801

[101] Cotter DG Schairer D Eichenfield L. Emerging therapies for atopic dermatitis: JAK inhibitors. J Am Acad Dermatol. (2018) 78:S53–62. 10.1016/j.jaad.2017.12.019 29248518

[102] Donnelly RP Sheikh F Dickensheets H Savan R Young HA Walter MR. Interleukin-26: An IL-10-related cytokine produced by Th17 cells. Cytokine Growth Factor Rev. (2010) 21:393–401. 10.1016/j.cytogfr.2010.09.001 20947410 PMC2997847

[103] Che KF Tengvall S Levänen B Silverpil E Smith ME Awad M. Interleukin-26 in antibacterial host defense of human lungs. Effects on neutrophil mobilization. Am J Respir Crit Care Med. (2014) 190:1022–31. 10.1164/rccm.201404-0689OC 25291379

[104] Che KF Kaarteenaho R Lappi-Blanco E Levänen B Sun J Wheelock Å. Interleukin-26 production in human primary bronchial epithelial cells in response to viral stimulation: modulation by Th17 cytokines. Mol Med. (2017) 23:1. 10.2119/molmed.2016.00064 28853490 PMC5653736

[105] Heftdal LD Andersen T Jæhger D Woetmann A Østgård R Kenngott EE. Synovial cell production of IL-26 induces bone mineralization in spondyloarthritis. J Mol Med. (2017) 95:779–87. 10.1007/s00109-017-1528-2 28365787

[106] Sheikh F Baurin VV Lewis-Antes A Shah NK Smirnov SV Anantha S. Cutting edge: IL-26 signals through a novel receptor complex composed of IL-20 receptor 1 and IL-10 receptor 2. J Immunol. (2004) 172:2006–10. 10.4049/jimmunol.172.4.2006 14764663

[107] Hör S Pirzer H Dumoutier L Bauer F Wittmann S Sticht H. The T-cell lymphokine interleukin-26 targets epithelial cells through the interleukin-20 receptor 1 and interleukin-10 receptor 2 chains. J Biol Chem. (2004) 279:33343–51. 10.1074/jbc.M405000200 15178681

[108] Hsu Y-H Chiu Y-S Chen W-Y Huang K-Y Jou I-M Wu P-T. Anti-IL-20 monoclonal antibody promotes bone fracture healing through regulating IL-20-mediated osteoblastogenesis. Sci Rep. (2016) 6:24339. 10.1038/srep24339 27075747 PMC4830982

[109] Poli C Augusto JF Dauvé J Adam C Preisser L Larochette V. IL-26 Confers proinflammatory properties to extracellular DNA. J Immunol. (2017) 198:3650–61. 10.4049/jimmunol.1600594 28356384

[110] Meller S Di Domizio J Voo KS Friedrich HC Chamilos G Ganguly D. T(H)17 cells promote microbial killing and innate immune sensing of DNA via interleukin 26. Nat Immunol. (2015) 16:970–9. 10.1038/ni.3211 26168081 PMC4776746

[111] Dambacher J Beigel F Zitzmann K De Toni EN Göke B Diepolder HM. The role of the novel Th17 cytokine IL-26 in intestinal inflammation. Gut (2009) 58:1207–17. 10.1136/gut.2007.130112 18483078

[112] Silverberg MS Cho JH Rioux JD McGovern DPB Wu J Annese V. Ulcerative colitis-risk loci on chromosomes 1p36 and 12q15 found by genome-wide association study. Nat Genet. (2009) 41:216–20. 10.1038/ng.275 19122664 PMC2652837

[113] Padua D Mahurkar-Joshi S Law IKM Polytarchou C Vu JP Pisegna JR. A long noncoding RNA signature for ulcerative colitis identifies IFNG-AS1 as an enhancer of inflammation. Am J Physiol Gastrointest Liver Physiol. (2016) 311:G446–57. 10.1152/ajpgi.00212.2016 27492330 PMC5076004

[114] Vandenbroeck K Cunningham S Goris A Alloza I Heggarty S Graham C. Polymorphisms in the interferon-gamma/interleukin-26 gene region contribute to sex bias in susceptibility to rheumatoid arthritis. Arthritis Rheum. (2003) 48:2773–8. 10.1002/art.11236 14558082

[115] Yeremenko N Noordenbos T Cantaert T van Tok M van de Sande M Cañete JD Disease-specific and inflammation-independent stromal alterations in spondyloarthritis synovitis. Arthritis Rheum. (2012) 65:174–85. 10.1002/art.37704 22972410

[116] LeBleu VS Taduri G O'Connell J Teng Y Cooke VG Woda C. Origin and function of myofibroblasts in kidney fibrosis. Nat Med. (2013) 19:1047–53. 10.1038/nm.3218 23817022 PMC4067127

[117] Ohnuma K Hatano R Aune TM Otsuka H Iwata S Dang NH. Regulation of pulmonary graft-versus-host disease by IL-26+CD26+CD4 T lymphocytes. J Immunol. (2015) 194:3697–712. 10.4049/jimmunol.1402785 25786689 PMC4568737

[118] Fickenscher H Pirzer H. Interleukin-26. Int Immunopharmacol. (2004) 4:609–13. 10.1016/j.intimp.2004.01.004 15120646

[119] Tengvall S Che KF Lindén A. Interleukin-26: an emerging player in host defense and inflammation. J Innate Immun. (2016) 8:15–22. 10.1159/000434646 26202572 PMC6738771

